# Proteomics and metabolomics analysis of hepatic mitochondrial metabolism in alcohol-preferring and non-preferring rats

**DOI:** 10.18632/oncotarget.22040

**Published:** 2017-10-25

**Authors:** Hao-Long Zeng, Qing Yang, Hongying Du, Huijun Li, Ying Shen, Taotao Liu, Xi Chen, Ghulam Mustafa Kamal, Qing Guan, Liming Cheng, Jie Wang, Fuqiang Xu

**Affiliations:** ^1^ Department of Laboratory Medicine, Tongji Hospital, Tongji Medical College, Huazhong University of Science and Technology, Wuhan, P.R. China; ^2^ State Key Laboratory of Magnetic Resonance and Atomic and Molecular Physics, Key Laboratory of Magnetic Resonance in Biological Systems, Wuhan Center for Magnetic Resonance, Wuhan Institute of Physics and Mathematics, Chinese Academy of Sciences, Wuhan, P.R. China; ^3^ College of Life Sciences, Wuhan University, Wuhan, P.R. China; ^4^ Key Laboratory of Environment Correlative Dietology, Ministry of Education, College of Food Science and Technology, Huazhong Agricultural University, Wuhan, P.R. China; ^5^ University of Chinese Academy of Sciences, Beijing, P.R. China; ^6^ Department of Chemistry, Government College University Faisalabad, Faisalabad, Pakistan; ^7^ Center for Excellence in Brain Science and Intelligence Technology, Chinese Academy of Sciences, Shanghai, P.R. China

**Keywords:** alcohol preference, liver mitochondria, quantitative proteomics, metabolomics, TCA cycle

## Abstract

Alcohol preference induced tolerance in humans and animals when their bodily functions adapt to compensate for the disruption caused by alcohol consumption. This was thought to be an important component of the genetic predisposition to alcoholism. To investigate the underlying mechanisms of hepatic metabolic tolerance during alcohol preference, the alcohol preferring and alcohol non-preferring rats were used in this study. The liver mitochondria were purified for comparative quantitative proteomics analysis, and the liver metabolite extracts were collected for metabolomics analysis. Our study identified 96 differentially expressed hepatic mitochondrial proteins that associated with alcohol preference, the further gene ontology and protein interaction network analysis suggest a down-regulation of amino acid metabolism and up-regulation of lipid metabolism. We found alcohol preference induced a series of enzymes decreased (e.g. SSADH and GABA-T) and several amino acids increased (e.g. glutamate and aspartate) in rat liver, indicating down-regulations of glutamate degradation occurred during alcohol preference. Most of these changes were due to the genetic differences between alcohol preferring and non-preferring animals. Furthermore, this study would provided new insights to further clarify the mechanisms of hepatic metabolic tolerance during alcohol preference.

## INTRODUCTION

Alcohol preferring or dependence is a complex condition involved in alterations of structures and functions in brain and liver, which was caused by a number of constitutional, environmental and genetic factors [1, 2]. As the primary tissue of alcohol metabolism, liver is one of the organs most likely to be damaged by alcohol drinking and it is susceptible to many of the pharmacological ramifications of alcohol abuse. In liver, excessive alcohol drinking could cause liver injury associated with profound impairments in hepatocellular regeneration [3]. Severity of alcohol usage is not specifically associated with the development of liver disease [4], which was probably caused by hepatic metabolic tolerance. Previous studies using the high alcohol preferring animals demonstrated that excessive alcohol intake resulted in sustained blood ethanol concentrations throughout the active period, leading to the development of metabolic tolerance [5]. This metabolic tolerance was likely involved during the alcohol abuse and chronic alcohol exposure. Changes of various biological processes in liver have been characterized by previous reports including regulations of oxidative phosphorylation, lipid metabolism and proteolytic systems in animal of alcohol preferring or chronic alcohol exposure [6–8]. However, the detailed adaptive alterations and the mechanisms involved in liver metabolic tolerance and alcohol preference or dependence remains poorly understood.

In order to investigate the mechanisms of liver metabolism during the alcohol preference, animal models are invaluable tools for elucidating the normal and abnormal functions. Most animals do not voluntarily consume sufficient amounts of alcohol to produce pharmacologically meaningful blood alcohol levels. Through selective breeding, the lines of high and low alcohol-consuming rats have been produced [9–11]. These models have been used to study the influence of genetic factors on the effects of alcohol and on alcohol drinking behavior [12]. However, most previous studies investigating the effect of alcohol preference on liver were always focused on the whole-tissue level [13, 14], while the structural and functional heterogeneity of liver made it become more necessary to enrich hepatic subcellular fractions for specific studies.

The rapid development of high-throughput technologies and computational frameworks enable the examination of biological systems in unprecedented detail [15]. Compared with genomics and transcriptomics, proteomics and metabolomics are located downstream of the entire system biology, representing the direct performer and final feedback of the overall function or state of the life system. During the study of proteomics and metabolomics, high resolution mass spectrometry (MS) could detect and quantify thousands of proteins and metabolites, combined with HPLC. Comparing with nuclear magnetic resonance (NMR) approach, the reproducibility is much lower, and sample pre-processing is more complicated. Thus the combination of MS and NMR have emerged as powerful and universal technologies for the global measurement of proteins and metabolites [16, 17].

In this study, the alcohol-preferring (AP) and non-alcohol-preferring (NAP) rats originated from Wistar colony [18] were utilized. The mitochondria cells in liver were purified for MS based comparative proteomics analysis, and the liver metabolites were extracted for NMR based metabolomics analysis. We successfully quantified 794 overlapped proteins among the biological replicates (Detected samples from different animals for every group) and technique replicates (Detected samples from the same animals for every group), from which 96 proteins were identified as regulated proteins involved in alcohol preference. The results showed that amino acid including tryptophan, glutamate, GABA metabolism was down-regulated, while lipid metabolism was found possibly up-regulated. The alcohol preference induced a series of enzymes including SSADH, GABA-T decreased and amino acids including glutamate and aspartate increased, indicating a down-regulation of glutamate degradation occurred. These results show the adaptive changes in liver mitochondria at protein and metabolite level, which would provide new insights to clarify the mechanisms of liver metabolic tolerance during alcohol preference.

## RESULTS

### Alcohol exposure

**Figure 1:**
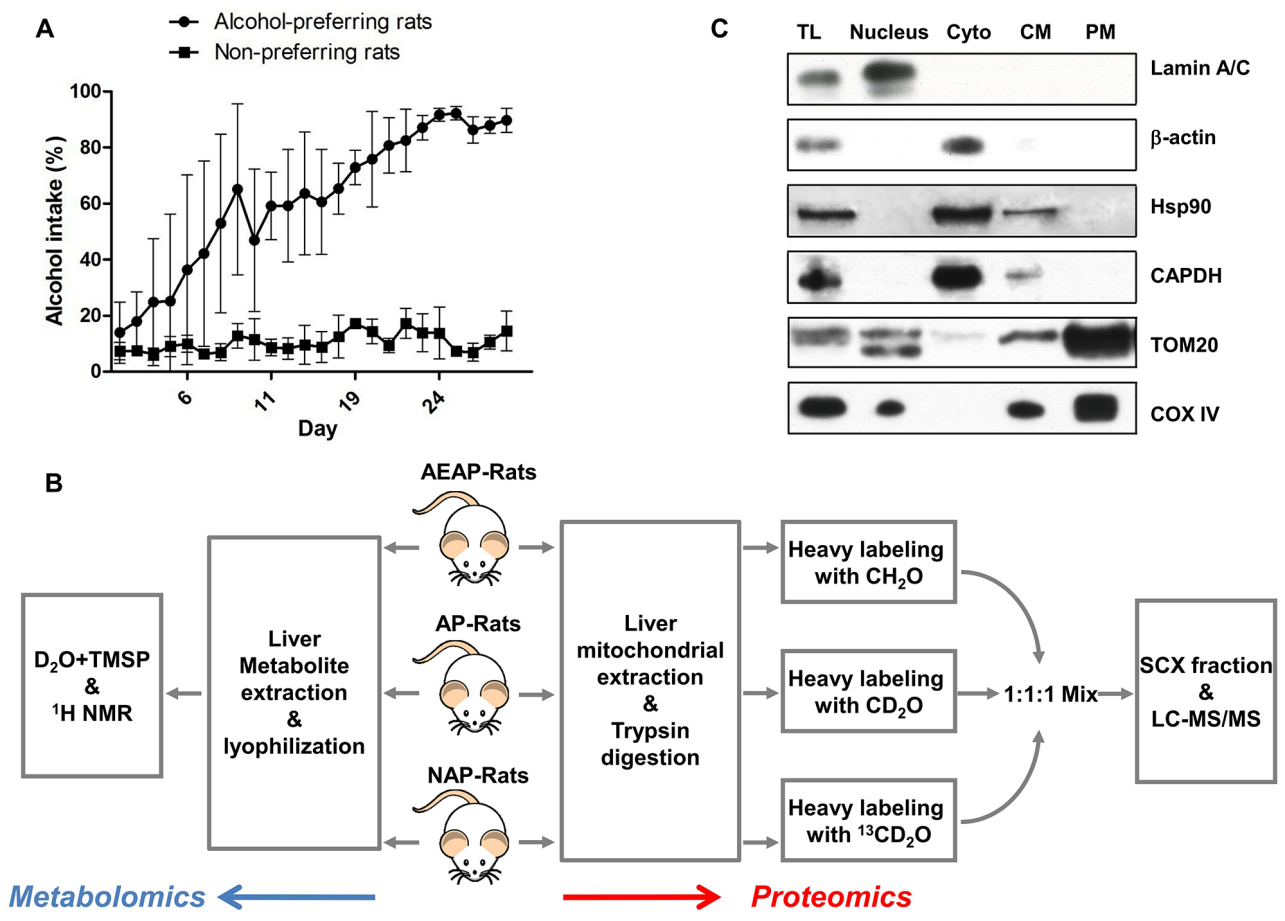
Experimental design **(A)**, alcohol consumption (%) was monitored for AP and NAP rats by alcohol/water two-bottle choice procedure. **(B)**, experimental flowchart. MS based quantitative proteomics (red arrow) and NMR based metabolomics (blue arrow) were applied. AP: alcohol preferring, NAP: alcohol non-preferring, AEAP: alcohol exposed alcohol preferring rat. **(C)**, purification of liver mitochondrial were confirmed by detecting mitochondria marker proteins TOM20 and COX VI, nuclear protein amin A/C, cytoplasm protein β-actin, GAPDH and Hsp90. TL: total lysate, Nucleus: nuclear fraction, Cyto: cytoplasm fraction, CM: crude mitochondria, PM: purified mitochondria.

Before the proteomics and metabolomics analysis of liver metabolism (Figure 1B), the alcohol consumption of the AEAP and NAP rats were monitored by the procedure of the alcohol-water two-bottle free choice training. Alcohol intake was found significantly increased for the AEAP rats after nearly a week training, and finally reached to a percentage of more than 80% alcohol consumption (Figure 1A), while the NAP rats only drink little alcohol during the whole training procedure, and their alcohol consumption was always around 10% (Figure 1A). Furthermore, the weight measurement was conduct by ourselves, and drops of liquid leakage from the bottle was unavoidable which would cause artificial error for measurement. Thus the AEAP rats almost only drink ethanol solution at the end of four weeks (5% alcohol consumption: 71.7 ± 18.4 g/kg) and NAP rats only drink water during the whole procedure (5% alcohol consumption: 7.3 ± 5.4 g/kg). The results demonstrated that the AEAP rats and NAP rats has been successfully trained for alcohol preference and non-preference after the method of alcohol-water free choice training.

### Mitochondrial purification

Considering the complexity of liver function and structure which comprised of multiple cell types, the mitochondria were purified for the subsequent proteomic analysis. The purification efficiency was assessed by detecting the mitochondrial maker protein Tom20 (Mitochondrial 20 kDa outer membrane protein) and Cox IV (Cytochrome c oxidase polypeptide IV). Results of the western blot showed a significant enrichment of Tom20 and Cox IV in the purified mitochondrial (PM) fraction. The nuclear protein Lamin A/C was only enriched in Nucleus fraction except for in the total lysate (TL), and the cytoplasm protein β-actin, Hsp90 and Gapdh were only enriched in cytoplasm (Cyto) fraction. These four proteins were almost undetected in the PM and crude mitochondria (CM) fraction (Figure 1C). This indicated that the strategy of mitochondrial purification was acceptable.

### Proteomics data overview

**Figure 2:**
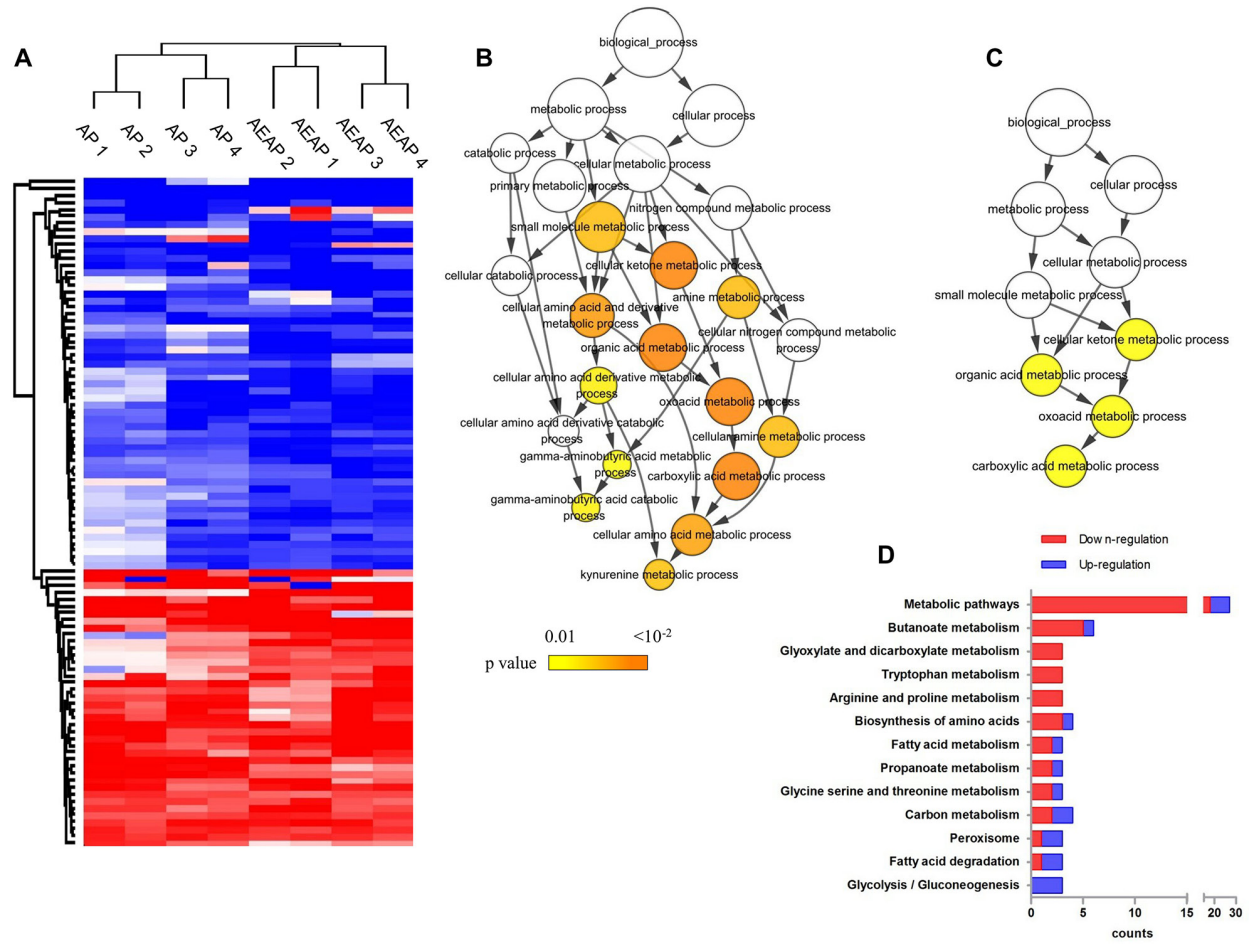
Proteomics data overview **(A)**, ninety-six differential proteins across the total four replicates in AP and AEAP rats were applied for clustering analysis. Replicates from AP rats and replicates from AEAP rats clustered respectively. Color represents quantitative ratio. **(B)**, the 56 down-regulated proteins in AP or AEAP rats were enriched in 13 GO terms. Color represents the enrichment significance. **(C)**, the 40 up-regulated proteins in AP or AEAP rats were enriched in 4 GO terms. Color represents the enrichment significance. **(D)**, the down- and up-regulated proteins were mapped in KEGG pathways. Pathways including at least 3 mapped proteins were considered.

From the four experiment replicates (two biological replicates times two technique replicates), a total of 1320 and 1318 proteins for AP *vs* NAP groups and AEAP *vs* NAP groups were successfully quantified, respectively. There were 794 and 792 proteins overlapped (Supplementary Figure 1). For these shared proteins, the Pearson correlation analysis [19] was performed to assess the reproducibility (Supplementary Figure 2). The Pearson correlation coefficient (PCC) was ˜0.70 for the technique replicates and ˜0.45 for the biological replicates for both of the AP *vs* NAP groups and AEAP *vs* NAP groups (Detail values in Supplementary Figure 2). To further extract the differentially expressed proteins, it was filtered that the proteins which changed at least 50% compared with the NAP rats in the datasets (Quantitative average ratio > 1.50 for up-regulation or < 0.67 for down-regulation). Finally, a total of 96 differential proteins was quantified (60 for AP *vs* NAP, 86 for AEAP *vs* NAP) (Supplementary Figure 1). The hierarchical clustering analysis was performed for these total 96 regulated proteins across all the replicates, and the alterations for AP rats and AEAP rats seems to be quite similar, but the replicates for AP rats or AEAP rats were still clustered together, respectively (Figure 2A). The final ratio of the quantified proteins for the following analysis was calculated as average of the four replicates. The all quantified proteins and differential proteins were listed in Supplementary Tables 1 and 2.

Considering most of the differential proteins changed consistently between AP *vs* NAP and AEAP *vs* NAP groups (Figure 2A), the total of 96 proteins was utilized for GO enrichment analysis to examine the biological processes involved during the alcohol preference in liver mitochondria. As shown in Figure 2B, the down-regulated proteins in AP or AEAP rats were significantly enriched among 13 terms (p < 0.01) which were mainly related to GABA catabolic process and kynurenine metabolic process. However, the up-regulated proteins were enriched among only four terms (p < 0.01) mainly involved in carboxylic acid metabolic process (Figure 2C). Compared with the up-regulation, down-regulation in AP or AEAP rats was found involved in more metabolic processes.

To find detailed metabolic pathways which related with alcohol preferring in rat liver, the 96 collected regulated proteins were submitted to the KEGG server (http://www.genome.jp/kegg/) for pathway mapping analysis. As shown in Figure 2D, similarly with the GO analysis, down-regulation by alcohol preference were dominant, and mainly including amino acid (Typtophan, arginine, proline) metabolism and other derivatives (butanoate, glyoxylate, propanoate) metabolism, while the up-regulation were mainly involved in fatty acid degradation and glycolsis / gluconeogenesis.

### Western blot analysis of the differential proteins

**Figure 3:**
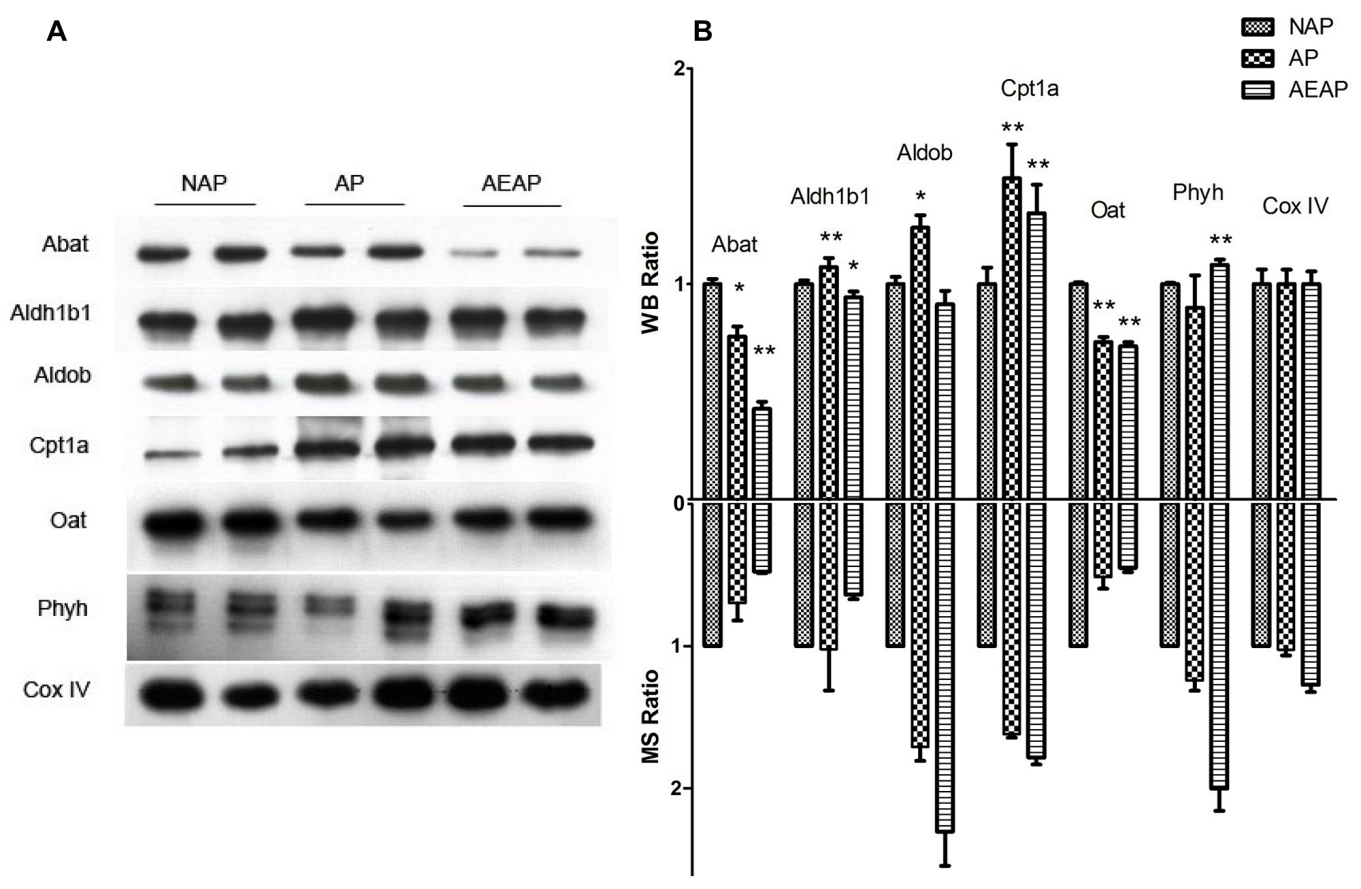
Western blot analysis of differential proteins **(A)**, a total of nine proteins were selected to be analyzed by western blot (WB) using β-actin as a loading control. **(B)**, comparison between WB ratios and MS ratios indicated that most of our WB results were consistent with the quantitative MS ratios. The two-tailed Student's *t*-test was applied. ^*^ p<0.5, ^**^ p<0.01, The group data shown are the average ± SEM.

As an approach to cross-check the reliability of quantitative proteomics data, the conventional western blot was usually utilized to assess the expression levels of several selected differential proteins. Here, a total of six metabolic enzymes were chosen for Western blot analysis, including two down-regulated proteins Abat and Oat, and four up-regulated proteins Aldh1b1, Aldob, Cpt1a and Phyh (Figure 3A). These proteins were reported of playing important roles in mitochondrial metabolism, and all found differentially expressed in AP and AEAP rats compared with NAP rats. Our results demonstrated that most of the changes identified by LC-MS/MS were consistent with those detected by the western blot (Figure 3B). The mitochondrial protein Cox IV was used as the loading control.

### Protein interaction network analysis

**Figure 4:**
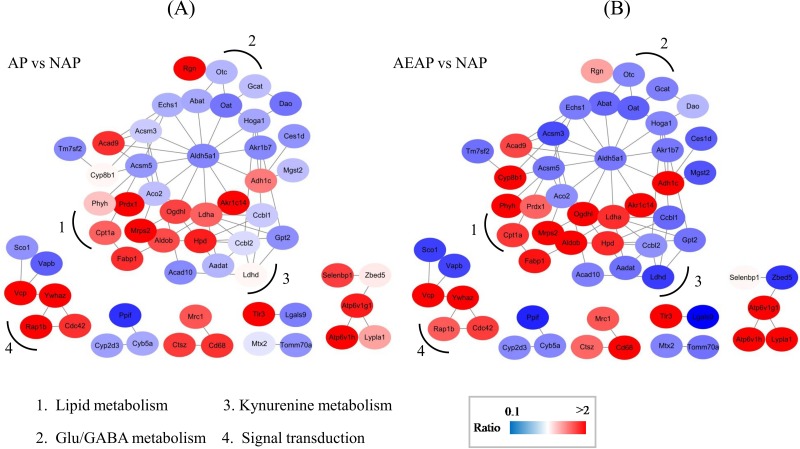
Metabolic network of regulated proteins in AP rats **(A)** and AEAP rats **(B)**. Proteins regulated in AP and AEAP rats were submitted to STRING 9.0 for network analysis. Sub-networks related to Lipid metabolism, kynurenine metabolism, Glu/GABA metabolism and Signal transduction were identified. The network includes 56 nodes and 78 edges. The node color represents the quantitative ratio.

Both of the GO and KEGG analysis indicated that several metabolic processes in the liver were probably related with the alcohol preference. To investigate the possible regulation network among these proteins, an interaction network analysis were performed by submitting the proteins to the STRING database. The interaction network that comprised of 56 proteins was established for AP *vs* NAP groups and AEAP *vs* NAP groups, respectively (Figure 4). To further analyze the network, these proteins were categorized into several pathway clusters, including lipid metabolism, glutamate / GABA metabolism, kynurenine metabolism and signal transduction according to the protein annotations in UniProt database (http://www.uniprot.org/). As shown in Figure 4, we found the clusters of glutamate/GABA metabolism and kynurenine metabolism seem to be down-regulated in both AP and AEAP rats, while the cluster of lipid metabolism showed up-regulated.

Moreover, it could be found that the tendency of the protein changes in AP and AEAP rats were quite consistent. Most of these proteins in the AEAP rats were changed much more than in the AP rats, with a very few exceptions like protein Rgn and Dao. The up-regulated proteins and down-regulated proteins in the network were well separated, which implied a possible co-regulation occurred in the alcohol preferring rats. More importantly, we found a key protein, Aldh5a1, a succinate - semialdehyde dehydrogenase (SSADH), that linked the down-regulated clusters and up-regulated clusters, might play a core role in the interaction network (Figure 4). It was down-regulated by 0.57 ± 0.11 and 0.53 ± 0.05 (Mean ± SD) fold in AP group and AEAP group, respectively, compared with NAP group. These results demonstrated that compared with the NAP rats, the alcohol preference leads to lots of alterations in the liver metabolic network, and the alterations were probably involved in a co-regulation surrounding the Aldh5a1 (SSADH).

### NMR based metabolomics

**Figure 5:**
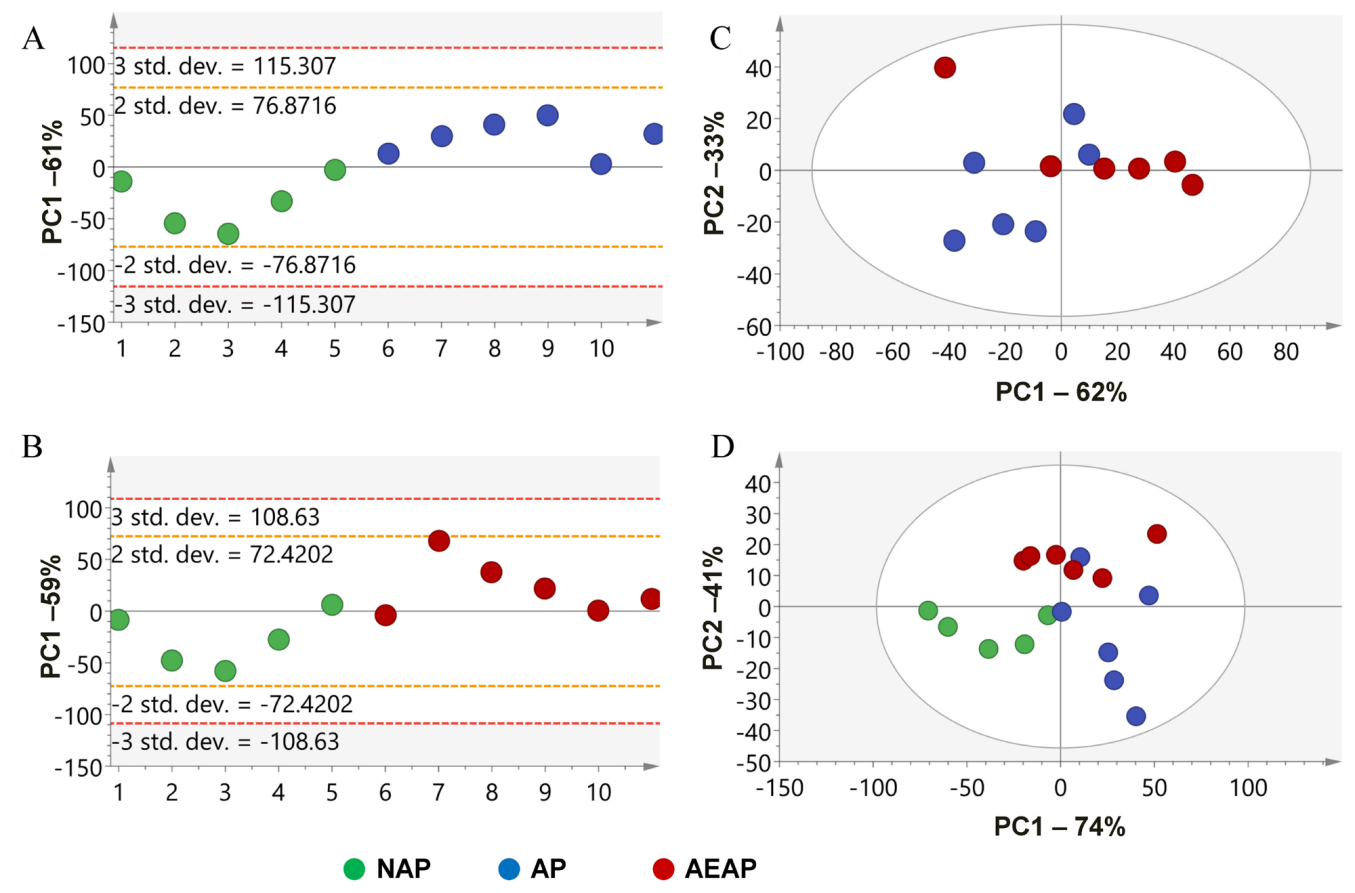
Principal component analysis (PCA) of the liver metabolite extracts for alcohol preferring and non-preferring rats **(A)** NAP vs. AP; **(B)** NAP vs. AEAP; **(C)** AP vs. AEAP; **(D)** all groups. Red: AEAP rats, Green: NAP rats, Blue: AP rats.

In order to test the changes of metabolites caused by the alterations of liver mitochondrial proteome with alcohol preference, the NMR based analysis was performed on the liver metabolite extracts from NAP, AP and AEAP rats. Using the normalized NMR data sets of every rat [20], PCA analysis was performed to generate an overview for group clustering for the AEAP, AP and NAP rats by using the SIMCA-P+ software. As shown in Figure 5, the principle components PC1, PC2 or PC3 among NAP, AP and AEAP rats were collected. The contributions of components were ˜60% or higher and played major roles in all the comparison groups. The samples with NAP and AP rats (Figure 5A), NAP and AEAP rats (Figure 5B) were both well separated, which means that the NAP rats have significantly different liver metabolite patterns compared with AP or AEAP rats, while the samples with AP and AEAP rats were almost mixed together (Figure 5C). These results were consistent with the animal preference (alcohol preferring or non-preferring).

**Figure 6:**
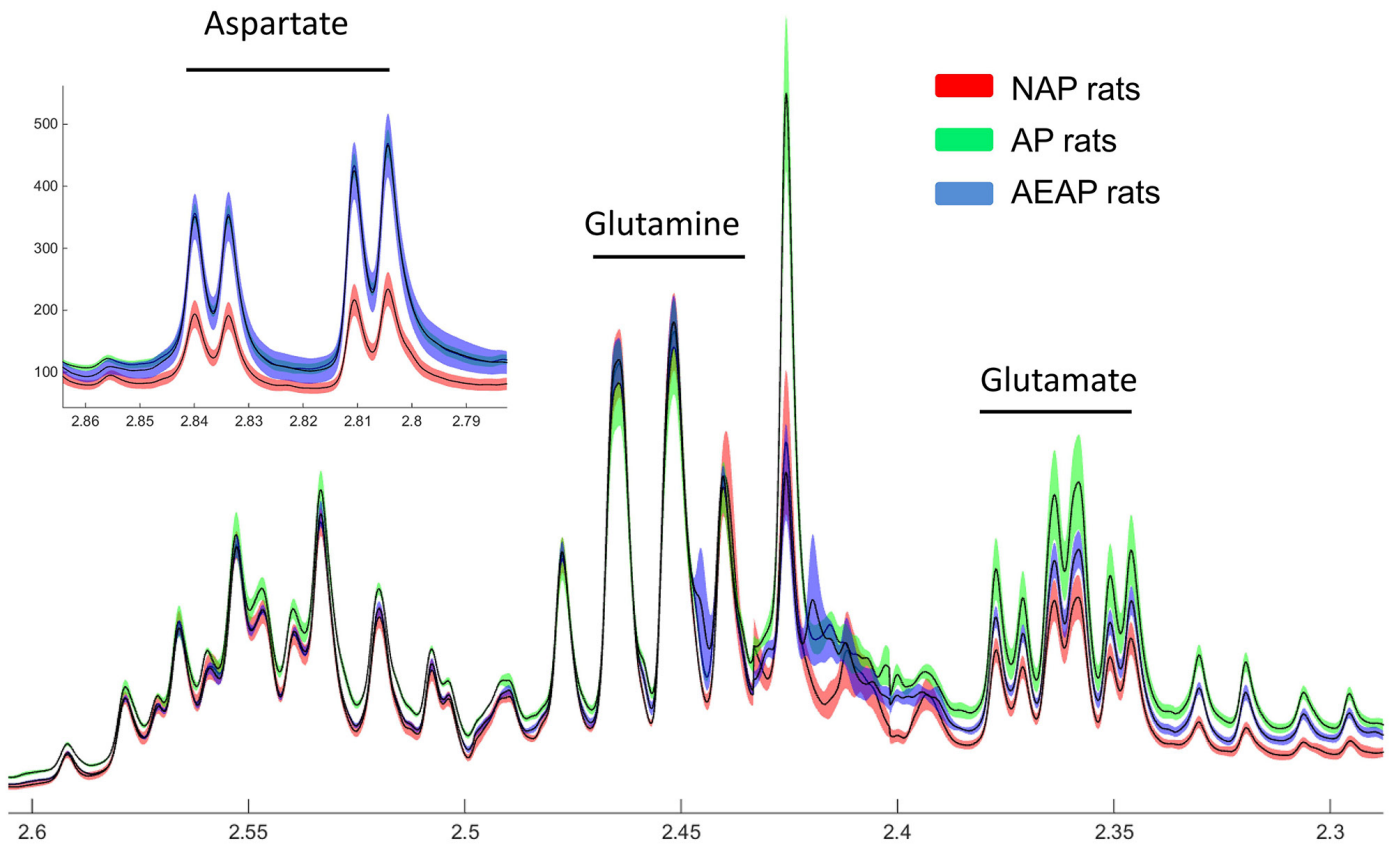
NMR spectrum of Glu, Gln and Asp NMR analysis of liver metabolite extracts in NAP, AP and AEAP rats. Red: NAP rats, Green: AP rats, Blue: AEAP rats, width of the color band represents error, shown as average ± SEM.

In terms of specific metabolites, the average concentrations of aspartate, glutamine and glutamate from the NMR spectra for NAP, AP and AEAP rats were calculated, which were obtained from the normalization of the spectra with the total area of the NMR spectrum [20]. The raw data of average and standard deviation of the three metabolites are calculated and displayed in Figure 6 (Draw with MATLAB). We found that aspartate and glutamate showed significant differentially expressions between rats of alcohol preference (AP & AEAP) and non - preference (NAP). While there was almost no difference in glutamine between these three kind of animals. Thus the genetic differences of alcohol preference seems to induce a much higher expressions of aspartate and glutamate in liver.

## DISCUSSION

To study the influence of alcohol preference on liver metabolism, the AP and NAP rats were utilized in the current study [5]. Results from the alcohol/water two-bottle free choice procedure (Figure 1A) showed that AP and NAP rats had become alcohol preference and non - preference, respectively.

Alcohol preference can induce tolerance in humans and animals when their bodily functions adapt to compensate for the disruption caused by alcohol consumption, especially for the hepatic metabolic tolerance, which was thought involved in the regulation of alcohol preference development [21]. The MS based quantitative proteomics and NMR based metabolomics approaches were combined to study the alterations of liver mitochondrial proteome and liver metabolites. Thus in the current study, liver metabolism was investigated from both mitochondrial protein and metabolite level for alcohol preference and non - preference.

At protein level, liver mitochondria were purified for quantitative proteomics analysis. Finally, a total of 96 differentially expressed proteins in AP or AEAP rats were identified (60 for AP *vs* NAP, 86 for AEAP *vs* NAP) (Supplementary Figure 2, Supplementary Table 2). The hierarchical analysis of these regulated proteins showed a very similar distribution between AP and AEAP rats (Figure 2A), indicating the four weeks alcohol exposure only induced few evident additional changes in liver metabolism, which was consistent with previous report [5].

Furthermore, the GO and KEGG analysis demonstrated a down-regulation of amino acid metabolic process including tryptophan (Trp) (Figure 2D), kynurenine (Figure 2B) and GABA (Figure 2B) *et al*., and a up-regulation of organic acid metabolic process including oxyacid, carboxylic acid (Figure 2C) and fatty acid (Figure 2D) *et al*.. Similar with these results, the interation networks analysis also suggested that glutamate/GABA metabolism and kynurenine metabolism showed downward co-regulated in both AP and AEAP rats, while the lipid metabolism showed up-regulated. These results implied the import roles for glutamate/GABA, kynurenine and lipid metabolism in liver for alcohol induced metabolic tolerance. Meanwhile, PCA analysis suggested a distinct pattern of metabolites in AP and AEAP rats compared with NAP rats, while almost no significant difference were found between AP and AEAP rats (Figure 5), which was also consistent with the metabolic analysis of aspartate and glutamate. The metabolic analysis was consistent with our previous proteomic results.

**Figure 7:**
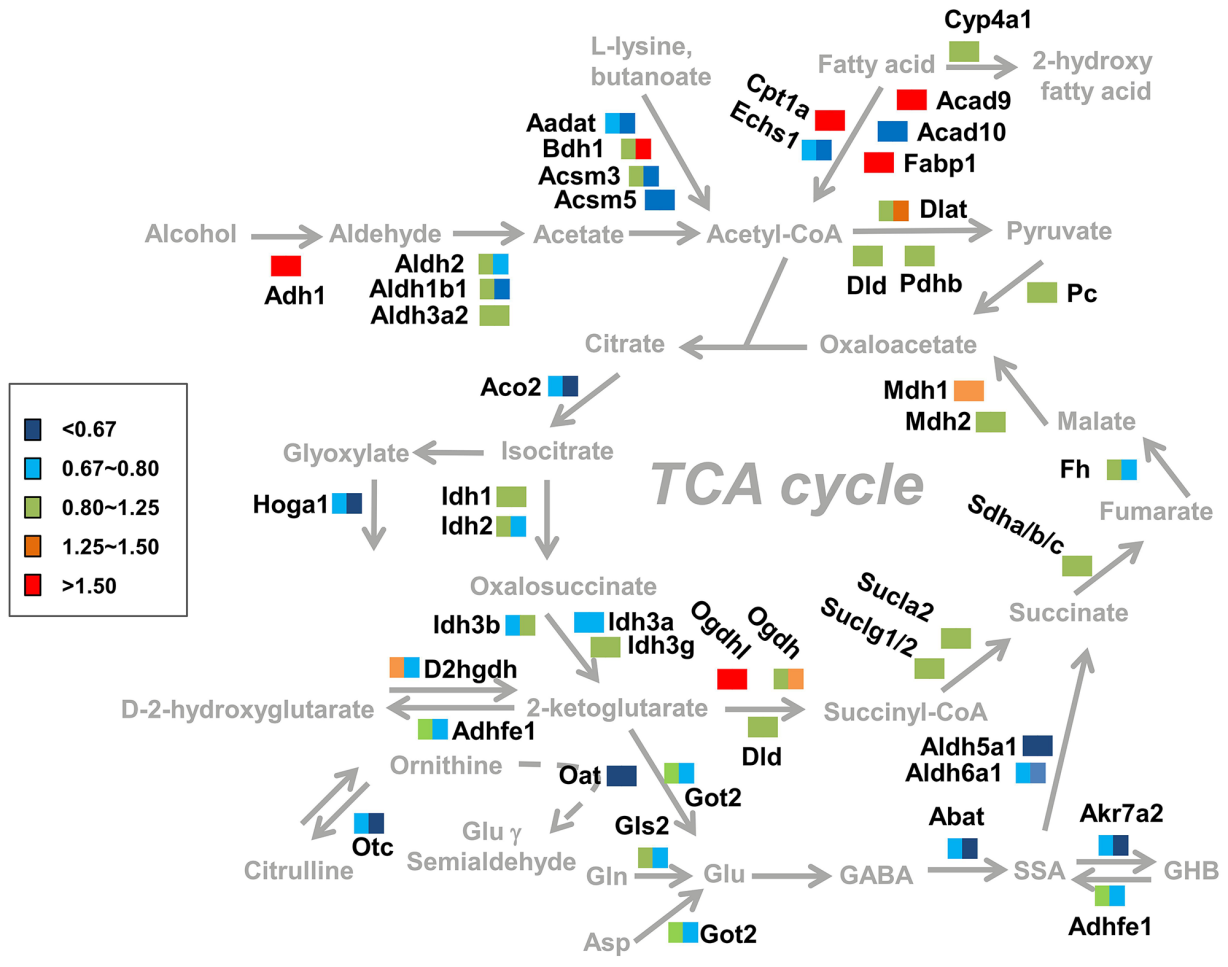
Summary of the changes in metabolic pathways observed in response to alcohol dependence Proteins involved in pathways of alcohol metabolism and TCA cycle were considered. The color rectangular box (including left square and right square) represent the quantitative ratio of protein changes in AEAP rats (left color area) and AP rats (fight color area).

To summary, these alterations at the level of both protein and metabolites were all associated with the alcohol preference, and a cellular pathway diagram surrounding cellular TCA cycle in liver was constructed including both protein and metabolite information according to our findings (Figure 7). Pathways of Trp-kynurenine, GABA-SSA and lipid metabolic pathway were changed for the alcohol preference, and were further discussed in more detail below.

### Trp-kynurenine metabolism

It has been reported that chronic alcohol exposure impairs One-Carbon metabolism, resulting in DNA hypomethylation in rat liver [22]. This is consistent with the down-regulation of liver amino acid metabolism, including Trp and kynurenine that suggested by the GO and KEGG analysis in our study (Figure 2B and 2D). As a precursor of 5-hydroxytryptamine (5-HT) which controls a variety of important functions in the central nervous system, Trp degradation was found inhibited during chronic alcohol intake in alcohol dependent subjects [23]. In our study, the GO analysis found that the metabolism of kynurenine, the main hepatic metabolic product of Trp degradation [23], showed tendency of down-regulation in AP or AEAP group (Figure 2B). Moreover, a sub-network involved in kynurenine metabolism was also identified which showed a downward co-regulation in AP and AEAP groups (Figure 4) in the protein interaction networks. Several enzymes involved in kynurenine metabolism like Aadat, Ccbl1, Ccbl2 were found decreased. These results demonstrated that a down-regulation of Trp-kynurenine metabolic process could be induced in liver of alcohol preferring rats.

### Glutamate-GABA-succinic semialdehyde metabolism

GABA, the degradation product of glutamate (Glu), is catabolized in the mitochondrial matrix through the GABA shunt. It was transaminated to succinic semialdehyde (SSA) followed by oxidation to succinate by the concerted actions of GABA transaminase Abat (GABA-T) and succinic semialdehyde dehydrogenase Aldh5a1 (SSADH), respectively [24]. Interestingly, it was found in our study that the enzymes involved in GABA-SSA metabolism including Abat, Aldh5a1, Aldh6a1 were decreased in the liver of AP rat (Figures 4 and 7), especially for Aldh5a1 - the succinic semialdehyde dehydrogenase, which was found possibly playing a core role in the regulation by our network analysis (Figure 4). In fact, adaptive changes in GABA system contributed to alcohol tolerance and preference has been confirmed [25]. Low cortical GABA levels was reported in both alcohol-dependent and hepatic encephalopathy patients, and decreased synthesis of GABA and increased synthesis of glutamate in plasma might be related to withdrawal symptoms of chronic alcohol intake [26, 27]. Consistently with these results at protein level, we found glutamate in liver of AP and AEAP rats were increased indeed compared with NAP rats by the NMR analysis, while glutamine was almost unchanged (Figure 6).

Additionally, several enzymes involved with another neurotransmitter, Gamma-hydroxy butyrate (GHB), were also found decreased in AP and AEAP rats, including Akr7a2 (SSAR) and Adhfe1 (HOT) (Figures 4 and 7). These two enzymes were responsible for converting SSA to GHB and transhydrogenating GHB and α-ketoglutarate to D-2-hydroxyglutarate and SSA, respectively. Exogenous administration of GHB was reported of exerting a number of pharmacological effects, including reduction of intensity of alcohol withdrawal syndrome and alcohol consumption in both laboratory animals and human alcoholics [28]. These results suggested the activity of GABA-SSA-GHB metabolism in liver may be regulated during alcohol preferring.

### Lipid metabolism

Bioactive fatty acid or lipid accumulation was early reported to be associated with alcohol abuse induced liver and brain degeneration [3]. One proposed mechanism is alcohol-induced elevation in concentrations of bioactive lipids that mediate apoptosis, lead to cell loss [29]. In our study, fatty acid metabolism was found possibly up-regulated (Figure 2D). Several involved proteins like Acad9, Fabp1 and Cpt1a was found increasing in AP and AEAP rats. Especially, the up-regulation of mitochondria enzyme Cpt1a (Figures 4 and 5), the carnitine palmitoyltransferase 1A, was reported to increase the rate of fatty acid β-oxidation and reduce triacylglycerol accumulation in liver [30, 31].

To summarize, the alterations of liver metabolism induced by alcohol preference was investigated by combining MS based proteomics and NMR based metabolic analysis. The down-regulation of glutamate, tryptophan metabolism and up-regulation of lipid metabolism in alcohol preference were identified, and a series of enzymes including SSADH, GABA-T were found decreased and amino acids including glutamate and aspartate were increased, indicating regulations of glutamate metabolism occurred. Most of these changes were due to the genetic differences between NAP and AP animals. Our study show the adaptive changes in liver mitochondrial metabolism, which would benefit for deeply clarify the mechanisms of liver metabolic tolerance for alcohol preference.

## MATERIALS AND METHODS

### Animals

All animal experiments were carried out according to the protocols provided by the Wuhan Institute of Physics and Mathematics, Chinese academy of Science (No. 00012092).

Twelve female AP rats and six female NAP rats of 4-5 weeks old were used in this study. The twelve AP rats were randomly divided into two different treatment groups: Group one (AP, n=6) was allowed free access to water only, group two (AEAP, n=6) was treated with 24 hours free-choice access to two bottles (polypropylene) containing tap water and 5% v/v alcohol solution for four weeks. In order to guarantee the alcohol concentration, the alcohol solution was refreshed every two days. The positions of the bottles were randomly changed every day to prevent the side preference, and the weight of ethanol intakes were daily recorded (9 am). Furthermore, two bottles free choice was also used to screen the NAP rats (Ethanol drinking ratio was lower than 20% [32]) similar with the method in the group of AEAP. The rats were housed in one animal per cage and on regular 12h/12h light-dark cycle at room temperature (22 ± 2°C), and their body weights were carefully monitored during the drinking period. The feeding pairs of animals were obtained from University of Melbourne (Prof. Andrew John Lawrence) with the permission from School of Medicine, Indiana University.

### Sample preparation

On the experimental day, rats were anesthetized with over-dose isoflurane, and livers were collected. One set of samples (˜500 mg per rat) were used for mitochondria purification and proteomics analysis, and the other set (˜100 mg per rat) was collected for NMR based metabolomics analysis.

For the analysis of mitochondria, a schematic flow of the LC-MS/MS based quantitative proteomics experiment by using triplex stable isotope dimethyl labeling was illustrated in Figure 1B. Comparative analyses were performed among AEAP, AP and NAP groups. To be more specific, equal weight of three liver samples from different rats in the same group were mixed together. Then the mitochondria were isolated by the density gradient centrifugation according to the previous works [33, 34], with a little modification. Briefly, freshly liver tissue were cut into tiny pieces in the isolation buffer containing with various protease and phosphatase inhibitors (250 mM sucrose, 5 mM MgCl_2_, 0.2 mM Na_3_VO_4_, 1 mM NaF and 50 mM Tris at pH = 7.4, with a protease inhibitor cocktail mixture (COMPLETE, Roche Applied Science)). The liver pieces were thoroughly rinsed with the isolation buffer and homogenized in the isolation buffer (1 mL). After filtration through a 100 mesh filter, the homogenate was centrifuged twice at 770 *g* for 10 min to remove the unbroken cells and nuclei. The supernatant was decanted and centrifuged at 15,000 g for 15 min to obtain the crude mitochondrial pellets. The pellets were washed and suspended in the ice-cold suspension buffer (200 mM mannitol, 50 mM sucrose, 1 mM EDTA, 0.5 mM EGTA, 0.2 mM NA_3_VO_4_, 1 mM NaF, and 10 mM Tri-HCl at pH = 7.4 with the protease inhibitor cocktail mixture) and were further purified on a 17 - 51% percoll density in an ultracentrifuge. The mitochondrial pellets in the interface between 42% and 51% were collected and stored at -80°C.

For the study of metabolites, the liver samples were extracted with the same protocol as our former studies [35, 36]. Here the procedure was briefly described. The samples were added into a 2 ml EP tube, and added HCl/methanol (80μL, 0.1M). Then the tissues were homogenized with Tissuelyser (Tissuelyser II, QIAGEN, German) for 1.5 min at a frequency of 20 Hz. Another 600 μL 60% ethanol (vol/vol) was added into the tubes and the mixture was homogenized again under the same condition. The mixture was centrifuged at 14,000 *g* for 15 min and the supernatant was collected. The whole extraction steps were repeated twice with 800 μL 60% ethanol. All the supernatants were collected together and desiccated with the centrifugal freeze-drying equipment (Thermo Scientific 2010, Germany). The dried product was stored at -80°C for further NMR studies.

### Extraction and digestion of mitochondrial proteins

The mitochondrial pellets from the percoll density gradient ultracentrifuge were suspended in lysis buffer (7 M urea, 2 M thiourea, 5mM DTT and the protease inhibitor cocktail). After sonication for 3 min (5 s intervals for every 2 s), the ultrasound assisted lysate was centrifuged at 20, 000 *g* for 20 min. The supernatant was collected, and concentration of the mitochondrial protein lysate was determined by the Bradford assay. The protein solution was reduced with 10 mM DTT at 37°C for 45 min, alkylated with iodoacetamide (30 mM) at room temperature for 45 min. Then the proteins were digested at pH 8.0 with 1:50 (w/w) trypsin (Promega, V5113) and incubated overnight at 37°C with shaking. The digested peptides were desalted using a SepPark C18 cartridge (Waters) and dried with a SpeedVac.

### Stable isotope dimethyl labeling and SCX fractionation

The desalted peptides were diluted with 0.1 M sodium acetate (pH = 6.0). As shown in Figure 1B, 4% (v/v) CH_2_O, CD_2_O and ^13^CD_2_O were added into the peptide samples from the AEAP rats, the AP rats and the NAP rats, respectively. Then sodium cyanoborohydride (NaBH_3_CN, 0.6 M) was added into these three kinds of solution for further incubation at room temperature for 1 h [37]. After that, the labeling reactions were quenched by adding 1% ammonium hydroxide, followed by 5% formic acid. At the end, these three samples were equally mixed and desalted prior to separation *via* strong cation exchange (SCX) chromatography.

For SCX fractionation, the mixed peptides were re-suspended in buffer (5 mM KH_2_PO_4_ and 20% acetonitrile, pH 2.7). The fractionation was performed on a polysulfoethy column (2.1 × 50 mm, 5 m × 200 Å) using a KCl gradient from 0.0 to 0.5 M. Eight fractions were collected and desalted with a C18 ZipTip (Minipore).

### LC-MS/MS and data processing

A Triple TOF 5600+ System coupled with an Ultra 1D Plus nano-liquid chromatography device (SCIEX, USA) was used for tandem MS analysis. Peptides were dissolved in 0.1% formic acid / 2% acetonitrile / 98% H_2_O, loaded into a C18 trap column (5 μm, 5 × 0.3 mm, Agilent Technologies) at a flow rate of 5 μL/min, and subsequently eluted from the trap column over the C18 analytic column (75 μm × 150 mm, 3 μm particle size, 100 Å pore size, Eksigent) at a flow rate of 300 nL/min in a 100 min gradient. The mobile phase consisted of two components: component A was 3% DMSO / 97% H_2_O with 0.1% formic acid, and component B was 3% DMSO / 97% acetonitrile with 0.1% formic acid. The information dependent acquisition (IDA) mode was used to acquire MS/MS data. Survey scans were acquired in 250 ms and 40 product ion scans were collected at 50 ms / per scan. The precursor ion range was set from m/z 350 to m/z 1500, and the product ion range was set from m/z 100 to m/z 1500. Tandem mass spectra were extracted by Peakview version 2.0 (SCIEX, USA).

### Western blot

Equivalent amounts of protein samples were uploaded and separated by 12% SDS-PAGE gels and then electro-transferred onto polyvinylidenedifluoride (PVDF) membranes (Millipore Crop, Atlanta, GA, US). The membranes were blocked with 5% skim milk for 1 h and then probed with specific primary antibodies at a concentration of 1:1000 (Aldob1, Aldh1b1, Phyh, Gapdh, Tom20, β-Actin, Hsp90 (all from ProteinTech Group); Abat and Oat (all from ABclonal, China)). Then, the membranes were incubated with respective horseradish peroxidase (HRP) - conjugated secondary antibodies at 1:10000 concentration for 1 h. The HRP was subsequently detected *via* ECL (Bia-Rad). The band intensity was measured using the Quantity One software package (Bio-Rad laboratories, version 4.6.2). The data were analyzed in GraphPad Prism 5 using two-tailed Student's *t*-test. Expression is reported relative to Cox IV and normalized to control group, and the densitometry results were expressed as the Mean ± SEM. The Differences were considered statistically significant at *p* < 0.05.

### NMR profiling

The lyophilized extracts were dissolved with 60 μL phosphate buffer (*p*H = 7.2, 60 μL, 120 mg/L 3 - (Trimethylsilyl) propionic - 2, 2, 3, 3, d_4_ acid sodium salt (TMSP, 269913-1G, Sigma-Aldrich) in D_2_O) and 540 μL double distilled water. The mixture was vortexed and centrifuged (10 min, 14489 g, 4°C). The supernatant (500 μL) from each sample was transferred to a 5 mm NMR tube for ^1^H-NMR analysis (298K, BurkerAvance III 600 MHz NMR spectrometer, Bruker Biospin, Germany). The ^1^H-NMR spectra were acquired with a standard WATERGATE pulse sequence [38]. The 90° pulse length was adjusted to about 10.1 μs for each sample and 128 transients were collected into 32 k data points over a spectral width of 20 ppm. In order to assign the metabolites in the ^1^H-NMR spectra, a series of two-dimensional (2D) NMR spectra were collected for a random sample: ^1^H-^1^H correlation spectroscopy (COSY), J-resolved spectroscopy (JRES), ^1^H-^1^H total correlation spectroscopy (TOCSY), ^1^H-^13^C heteronuclear single quantum correlation (HSQC), and ^1^H-^13^C heteronuclear multiple bond correlation (HMBC) 2D NMR spectra.

The phases of ^1^H-NMR spectra were manually corrected and the baseline distortion was completed in TOPSPIN (version 2.0, Bruker Biospin). The whole batch of spectra was aligned references to TMSP signal. The peak alignment and peaks extraction were completed with the home made software NMRSpec [39]. The areas of peaks were normalized to the total sum of the peak areas (between 0.80 ppm and 4.30 ppm) to compensate for sample concentration differences. The PCA (Principal component analysis) of the normalized NMR data sets were carried out using the SIMCA-P+ software package (version 11.0, Umetrics, Sweden), and the statistical analysis (Figure 6) were conducted in MATLAB (Version, 2014a).

### Bioinformatics

Biological Networks Gene Ontology (BiNGO) 3.03 was used to calculate the gene ontology (GO) term enrichment of significantly up- or down-regulated proteins (defined as quantitative ratio > 1.50 or < 0.67) and determine significantly under- and over-represented functional GO categories. The Cytoscape network visualization platform (http://www.cytoscape.org/) implementing the latest release of the BiNGO plug - in was used to identify proteins that were annotated on the basis of biological process categories. The analysis was conducted using the default BiNGO Rattus database. Statistical significance was determined by means of hypergeometric analysis, followed by Benjamini and Hochberg's false discovery rate correction [40]. The intracellular pathway analysis was performed by using the KEGG Pathway database *via* the KEGG automatic annotation server (http://www.genome.jp/kegg/) [41]. The differentially expressed proteins matched in the KEGG Pathway database were counted and processed by Microsoft office excel.

For protein interaction network analysis, the differential proteins based on the quantified MS results were submitted to STRING 9.0 (the Search Tool for the Retrieval of Interacting Genes/Proteins) to qualify the physical and functional interactions of these proteins. The proteins and their interactions were then uploaded to Cytoscape (version 2.8.3) for data visualization.

## SUPPLEMENTARY MATERIALS FIGURES AND TABLES







## Abbreviations

MS: mass spectrometry
NMR: nuclear magnetic resonance
AP: alcohol-preferring
NAP: non-alcohol-preferring
