# Supplementary material for: Proteomics and metabolomics analysis of hepatic mitochondrial metabolism in alcohol-preferring and non-preferring rats
Source: Oncotarget. 2017 Oct 25;8(60):102020–32. doi: 10.18632/oncotarget.22040 (PMC5731932; doi:10.18632/oncotarget.22040)
Supplement: Supplementary file 1 [file oncotarget-08-102020-s001.pdf]

Proteomics and metabolomics analysis of hepatic mitochondrial metabolism in alcohol-preferring and non-preferring rats

SUPPLEMENTARY MATERIALS

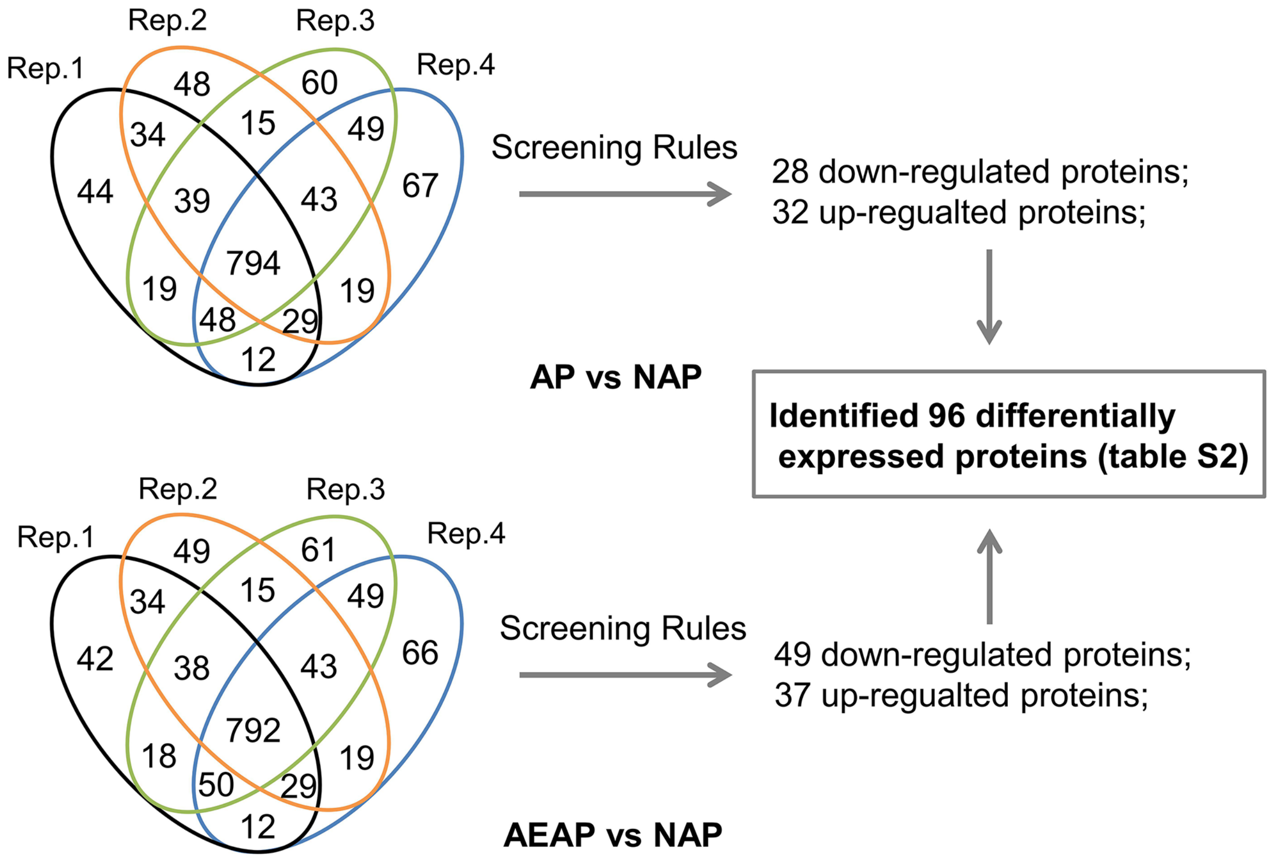

Supplementary Figure 1: Ninety six mitochondrial proteins were identified as differential proteins for AP or AEAP rats. A total of 1320 and 1318 proteins for the AP *vs* NAP groups and AEAP *vs* NAP groups was successfully quantified, respectively from the four experiment replicates (two biological replicates and each comprised of two technique replicates). There were 794 and 792 proteins overlapped. The differentially expressed proteins were filtered by rules that quantitative average ratio > 1.50 for up-regulation or <0.67 for down-regulation.

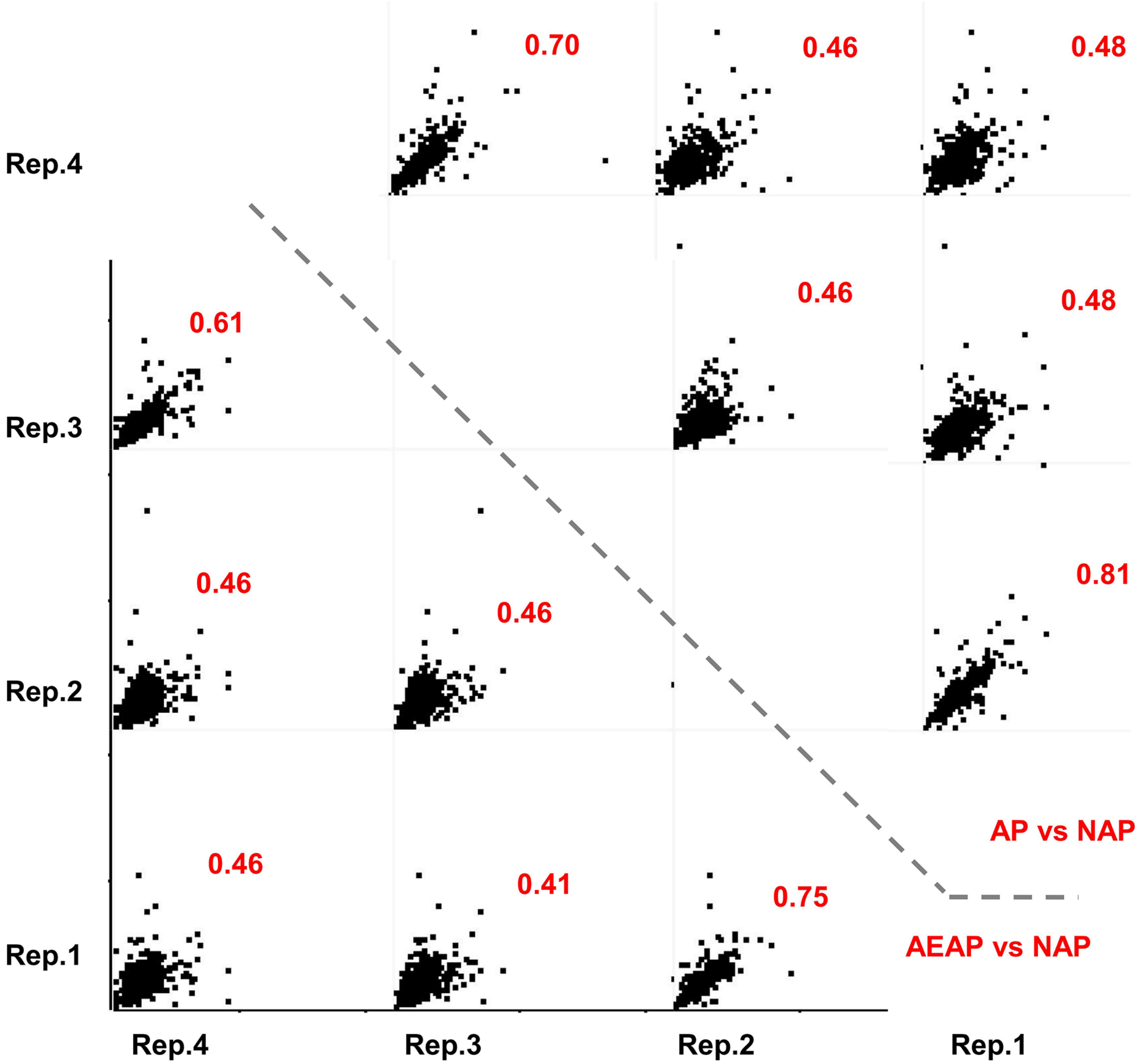

Supplementary Figure 2: Correlation analysis of all the four replicates for AP *vs* NAP groups and AEAP *vs* NAP groups. Pearson correlation analysis was performed to assess the reproducibility. Pearson correlation coefficient was ~0.70 for the technique replicates and ~0.45 for the biological replicates for both of the AP *vs* NAP group and AEAP *vs* NAP group.

Supplementary Table 1: Summary of quantitated protein. A total of 794 proteins were simultaneously quantified in all the replicates (two biological replicates times two technique replicates) in the study.

See Supplementary File 1

Supplementary Table 2: Summary of regulated protein. Ninety-four proteins were found differentially expressed in liver mitochondria of the AP or AEAP rats compared with NAP rats.

See Supplementary File 2
